# Supplementary material for: Disruptive mutations in TANC2 define a neurodevelopmental syndrome associated with psychiatric disorders
Source: Nat Commun. 2019 Oct 15;10:4679. doi: 10.1038/s41467-019-12435-8 (PMC6794285; doi:10.1038/s41467-019-12435-8)
Supplement: Supplementary file 1 — Supplementary Information [file 41467_2019_12435_MOESM1_ESM.pdf]

Supplementary Information

Disruptive mutations in *TANC2* define a neurodevelopmental syndrome associated with psychiatric disorders

Guo et al.

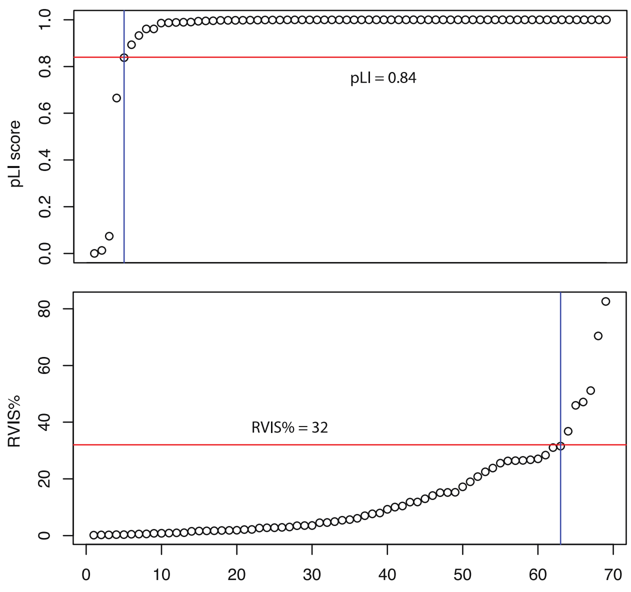

Supplementary Figure 1 Distribution of pLI scores and RVIS percentile of genes with *de novo* LGD mutations in SSC and ASC cohorts for genes that reach significance (FDR < 0.05) for an excess of *de novo* mutations based on the NDD meta-analysis (Coe et al., *Nat Genet*, 2019).

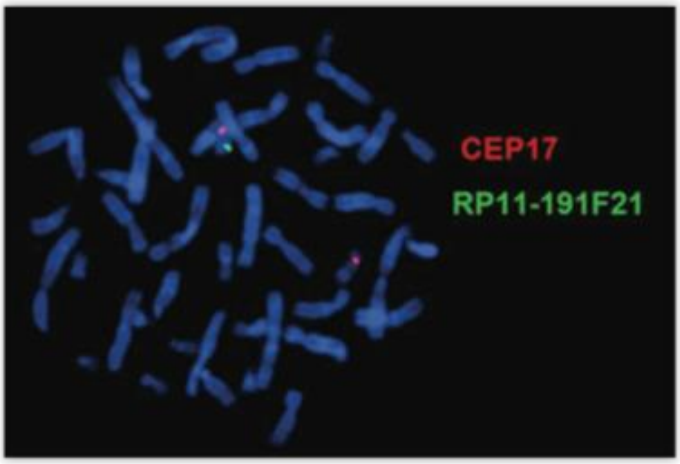

Supplementary Figure 2 One metaphase spread showing a heterozygous deletion at BAC RP11-191F21 by FISH performed to detect the mosaic status of the father in family CF. In total, we analyzed 150 mitoses and observed heterozygous deletions of BAC RP11-191F21 for 9/150 or 6% of cells.

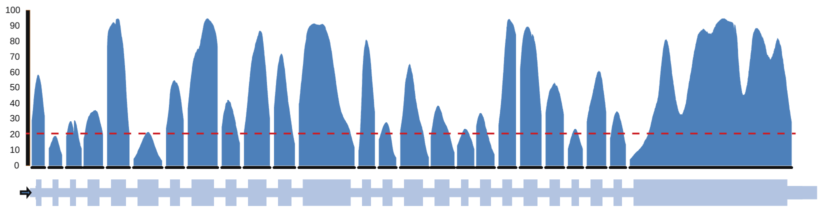

Supplementary Figure 3 Mean coverage of the coding regions of *TANC2* in ExAC WES data. The overall mean coverage is 49.28, and the majority of the coding nucleotide sites have a mean coverage over 20× (red line).

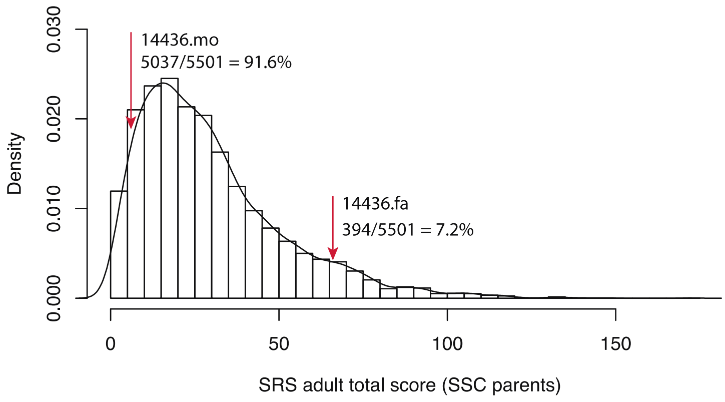

Supplementary Figure 4 Distribution of adult SRS score for all parents in SSC families. The SRS score of the father (SS2.fa) in family SS2 is located at the top 7.2 percentile.

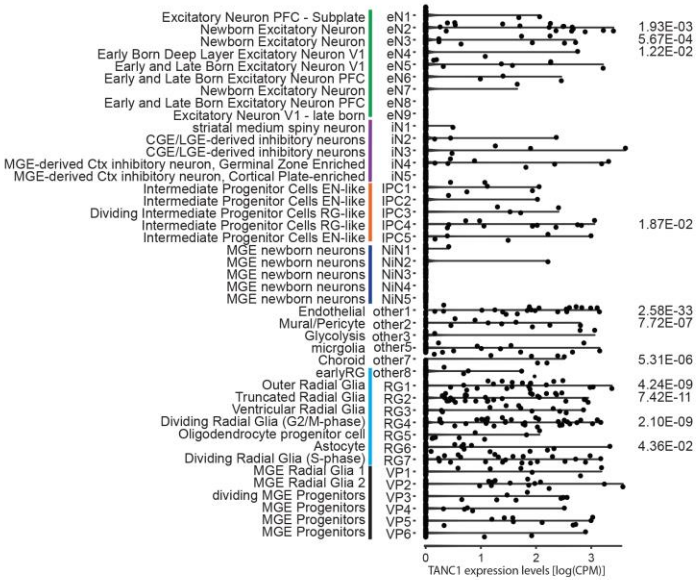

Supplementary Figure 5 Violin plot showing *TANC1* expression across the major cell types identified in the single-cell RNA-seq. Samples are ordered according to the average expression level across single cells of each type, and p-value represents Bonferroni-corrected p-value quantified using Wilcoxon rank sum test. n.s. – p > 0.05. Underlying data are provided as a Source Data file.

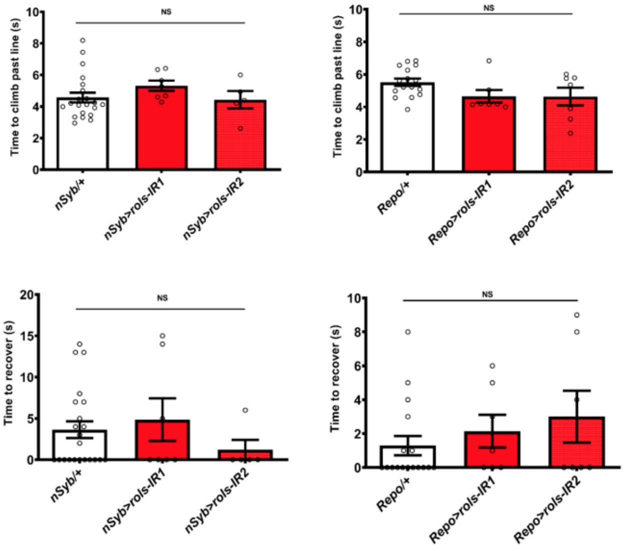

Supplementary Figure 6 Neuronal or glial knockdown of *rols* does not affect climbing nor induce bang-sensitivity phenotypes. Neuronal-specific (nSyb-Gal4) knockdown of *rols* does not affect climbing (top left) or bang-sensitivity (bottom left). nSyb/+, n=21; nSyb>rols-IR1, n=5; nSyb>rols-IR1, n=7. Glial-specific (Repo-Gal4) knockdown of *rols* does not affect climbing (top right) or bang-sensitivity (bottom right). nSyb/+, n=16; nSyb>rols-IR1, n=7; nSyb>rols-IR1, n=7. N.S, not significant. Error bars represent SEM.

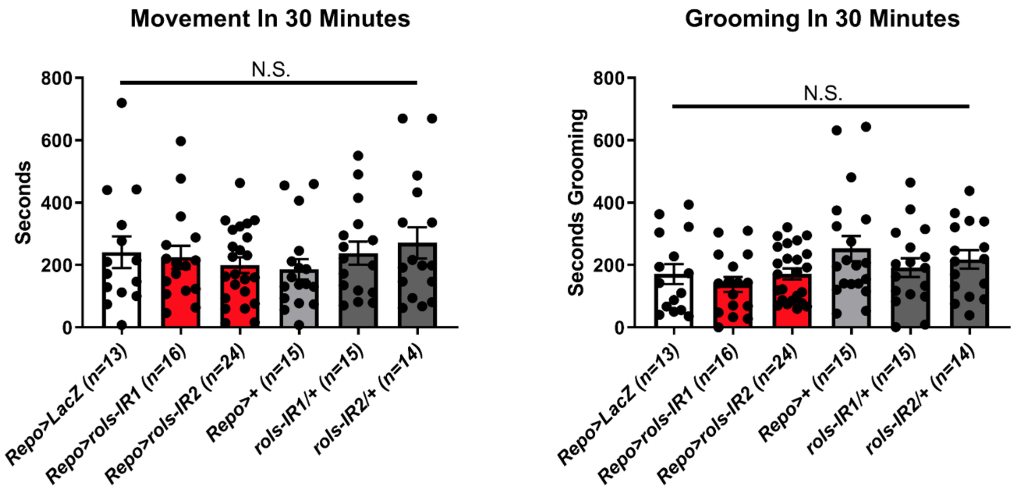

Supplementary Figure 7 Glial knockdown of *rols* does not affect locomotion or grooming behavior. Glial-specific (Repo-Gal4) knockdown of *rols* (IR1, n=16; IR2, n=24) does not change locomotion (left) or grooming (right) behavior compared to controls (LacZ, n=13; +, n=15; IR1/+, n=15; IR2/+, n=14). N.S, not significant. Error bars represent SEM. Underlying data are provided as a Source Data file.

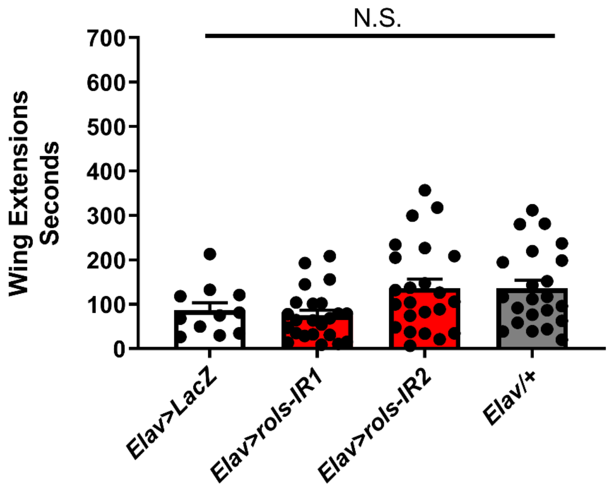

Supplementary Figure 8 Neuronal knockdown of *rols* does not affect courtship behavior. Neuronal-specific (Elav-Gal4) knockdown of *rols* (IR1 and IR2, n=23) does not change wing extension behavior compared to controls (LacZ, n=10; +, n=23). N.S, not significant. Error bars represent SEM.

Supplementary Table 1. WES coverage and estimated *de novo* mutation rate of cohorts with *TANC2* LGD mutations.

Patient ID	cohort	cohort size	cohort WES coverage	overall *de novo* mutation rate in the exome region	synonymous *de novo* mutation rate in the exome region
NN1.p1, NN2.p1, NN3.p1	Nijmegen	4500	~120x	1.44	0.289
LG.p1	Leipzig	180	124x	~ 1 per trio	na
HU1.p1	BCM	8910	>100x	1-2 in trios	na
HU2.p1	BCM	8910	>100x	1-2 in trios	na
AN.p1	Amsterdam	277	~100x	~ 1 per trio	na
PF.p1	Paris	651	79x	~ 2 per trio	na
MA.p1	Melbourne	209	52x	0.97	0.39
SS1.p1, SS2.p1	SSC	2508	89.2x	1.08	0.256

Supplementary Table 2. Detailed annotation for five *TANC2 de novo* missense mutations (NM_025185).

Sample.ID	PubMed.ID	Disorder	Validation	AAChange.refGene	CADD (1.3)	SIFT	Polyphen2	Mutation Taster	LRT	PROVEAN	MetaSVM	MetaLR	M-CAP	fathmm-MKL	GERP++	ExAC ALL
SS3.p1(13573.p1)	25363768	ASD	unknown	c.A5066G:p.H1689R	8.3	T	P	D	D	D	T	T	T	D	5.06	.
TU.p1(03C21418)	28191889	ASD	Y	c.G2882A:p.R961Q	29.3	D	D	D	D	N	T	T	T	D	5.78	3.57E-05
FS.p1(4047-1)	24463507	SCZ	Y	c.C2381T:p.A794V	34	D	D	D	D	D	D	D	D	D	5.62	.
NN4.p1(deLigt42)	23033978	ID	Y	c.C2278T:p.R760C	33	D	D	D	D	D	T	D	D	D	5.8	.
CC2.p1(M15225)	this study	ASD	Y	c.G2264A:p.R755H	32	D	D	D	D	D	D	D	D	D	5.8	3.53E-05

Supplementary Table 3. Biological interpretations of the cell clusters.

Cluster name	Interpretation	Cluster name (Science 2017, PMID: 29217575)
eN1	Excitatory Neuron PFC - Subplate	EN-PFC-1
eN2	Newborn Excitatory Neuron	nEN-early-2
eN3	Newborn Excitatory Neuron	nEN-late
eN4	Early Born Deep Layer Excitatory Neuron V1	EN-V1-1
eN5	Early and Late Born Excitatory Neuron V1	EN-V1-2
eN6	Early and Late Born Excitatory Neuron PFC	EN-PFC-2
eN7	Newborn Excitatory Neuron	nEN-early-1
eN8	Early and Late Born Excitatory Neuron PFC	EN-PFC-3
eN9	Excitatory Neuron V1 - late born	EN-V1-3
iN1	striatal medium spiny neuron	IN-STR
iN2	CGE/LGE-derived inhibitory neurons	IN-CTX-CGE-1
iN3	CGE/LGE-derived inhibitory neurons	IN-CTX-CGE-2
iN4	MGE-derived Ctx inhibitory neuron, Germinal Zone Enriched	IN-CTX-MGE-1
iN5	MGE-derived Ctx inhibitory neuron, Cortical Plate-enriched	IN-CTX-MGE-2
IPC1	Intermediate Progenitor Cells EN-like	IPC-nEN-1
IPC2	Intermediate Progenitor Cells EN-like	IPC-nEN-2
IPC3	Dividing Intermediate Progenitor Cells RG-like	IPC-div-1
IPC4	Intermediate Progenitor Cells RG-like	IPC-div-2
IPC5	Intermediate Progenitor Cells EN-like	IPC-nEN3
NiN1	MGE newborn neurons	nIN-1
NiN2	MGE newborn neurons	nIN-2
NiN3	MGE newborn neurons	nIN-3
NiN4	MGE newborn neurons	nIN-4
NiN5	MGE newborn neurons	nIN-5
Other1	Endothelial	Endothelial
Other10	Unknown4	Unk4
Other2	Mural/Pericyte	Mural
Other3	Glycolysis	Glyc
Other4	Unknown1	Unk1
Other5	Microglia	Microglia
Other6	Unknown3	Unk3
Other7	Choroid	Choroid
Other8	earlyRG	RG-early
Other9	Unknown2	Unk2
RG1	Outer Radial Glia	oRG
RG2	Truncated Radial Glia	tRG
RG3	Ventricular Radial Glia	vRG
RG4	Dividing Radial Glia (G2/M-phase)	RG-div-1
RG5	Oligodendrocyte progenitor cell	OPC
RG6	Astrocyte	Astrocyte
RG7	Dividing Radial Glia (S-phase)	RG-div-2
VP1	MGE Radial Glia 1	MGE-RG-1
VP2	MGE Radial Glia 2	MGE-RG-2
VP3	dividing MGE Progenitors	MGE-div
VP4	MGE Progenitors	MGE-IPC-1
VP5	MGE Progenitors	MGE-IPC-2
VP6	MGE Progenitors	MGE-IPC-3

Supplementary Table 4. Statistical results of the enrichment analysis for the clusters.

types	p-value	odds ratio	average expression
eN1	2.65E-17	0.605911998	1.980960684
eN2	1	0.064387121	0.89970734
eN3	1.12E-05	-0.352934522	0.615976682
eN4	4.74E-21	0.526252077	1.786841678
eN5	2.75E-09	0.430318321	1.411698107
eN6	0.000313906	0.423932611	1.359380885
eN7	0.032777195	0.406215662	1.427132837
eN8	1	0.243414012	1.207592008
eN9	0.001485718	0.417596772	1.390104616
iN1	1	-0.095929825	0.86392943
iN2	1	-0.096997281	0.85952813
iN3	1	0.000857232	0.970491913
iN4	0.00032081	-0.432533419	0.593143958
iN5	1	-0.034187779	0.878874442
IPC1	1	0.106986883	0.987555549
IPC2	0.000202366	-0.7935619	0.372082238
IPC3	1	-0.232439181	0.582517214
IPC4	1	-0.040470389	0.907448871
IPC5	1	-0.387782449	0.577323472
NiN1	1	-0.271073194	0.680650637
NiN2	1.66E-07	-1.031967179	0.305894038
NiN3	1	-0.141385874	0.681444316
NiN4	0.001289258	-1.930915223	0.106404522
NiN5	0.002292706	-0.814452603	0.415607929
other1	0.026530563	-1.082650068	0.27448574
other10	3.44E-10	0.590773966	2.001918784
other2	0.048293965	-1.541025798	0.130955407
other3	1	-0.135819245	0.843198809
other4	1	0.021027965	0.764688125
other5	4.92E-12	-2.036428753	0.027881711
other6	1	0.683837335	0.618114291
other7	1	-0.316735536	0.252654976
other8	0.268738379	-0.488747298	0.517523569
other9	1	0.191867447	0.825972969
RG1	4.30E-05	0.480582955	1.581015434
RG2	3.69E-07	0.438944105	1.563673082
RG3	1	0.05231004	0.981299035
RG4	1	0.1731146	1.132826532
RG5	0.108009649	0.36476987	1.500957941
RG6	1	0.255569388	1.213053654
RG7	1	0.131688905	1.028528538
VP1	1	0.15013378	1.050027094
VP2	0.075462559	-0.449643646	0.500654997
VP3	4.04E-05	-1.14119613	0.238111107
VP4	0.044694632	-0.773405111	0.414618405
VP5	0.012655565	-0.647730844	0.428052313
VP6	0.001740652	-1.035265132	0.20128953
	1	-0.20447906	0.762646251
