# Supplementary material for: Disruptive mutations in TANC2 define a neurodevelopmental syndrome associated with psychiatric disorders
Source: Nat Commun. 2019 Oct 15;10:4679. doi: 10.1038/s41467-019-12435-8 (PMC6794285; doi:10.1038/s41467-019-12435-8)
Supplement: Supplementary file 6 — Reporting Summary [file 41467_2019_12435_MOESM6_ESM.pdf]

nature research

Corresponding author(s): Evan E. Eichler & Alessandra Murgia

Reporting Summary

Nature Research wishes to improve the reproducibility of the work that we publish. This form provides structure for consistency and transparency in reporting. For further information on Nature Research policies, see Authors & Referees and the Editorial Policy Checklist.

Statistical parameters

When statistical analyses are reported, confirm that the following items are present in the relevant location (e.g. figure legend, table legend, main text, or Methods section).

n/a	Confirmed	
☐	☒	The exact sample size (n) for each experimental group/condition, given as a discrete number and unit of measurement
☐	☒	An indication of whether measurements were taken from distinct samples or whether the same sample was measured repeatedly
☐	☒	The statistical test(s) used AND whether they are one- or two-sided<br>*Only common tests should be described solely by name; describe more complex techniques in the Methods section.*
☐	☒	A description of all covariates tested
☐	☒	A description of any assumptions or corrections, such as tests of normality and adjustment for multiple comparisons
☐	☒	A full description of the statistics including central tendency (e.g. means) or other basic estimates (e.g. regression coefficient) AND variation (e.g. standard deviation) or associated estimates of uncertainty (e.g. confidence intervals)
☐	☒	For null hypothesis testing, the test statistic (e.g. F, t, r) with confidence intervals, effect sizes, degrees of freedom and P value noted<br>*Give P values as exact values whenever suitable.*
☒	☐	For Bayesian analysis, information on the choice of priors and Markov chain Monte Carlo settings
☒	☐	For hierarchical and complex designs, identification of the appropriate level for tests and full reporting of outcomes
☒	☐	Estimates of effect sizes (e.g. Cohen's d, Pearson's r), indicating how they were calculated
☐	☒	Clearly defined error bars<br>*State explicitly what error bars represent (e.g. SD, SE, CI)*

Our web collection on statistics for biologists may be useful.

Software and code

Policy information about availability of computer code

Data collection	N/A
Data analysis	We used the denovolyzeR and CH models to estimate the enrichment of TANC2 de novo mutations in NDD patients. The denovolyzeR model was published (PMID: 26439716) and described here http://denovolyzer.org/. The CH model was published and described in O'Roak et al. 2012 Science (PMID: 23160955).

For manuscripts utilizing custom algorithms or software that are central to the research but not yet described in published literature, software must be made available to editors/reviewers upon request. We strongly encourage code deposition in a community repository (e.g. GitHub). See the Nature Research guidelines for submitting code & software for further information.

Data

Policy information about availability of data

All manuscripts must include a data availability statement. This statement should provide the following information, where applicable:

- Accession codes, unique identifiers, or web links for publicly available datasets
- A list of figures that have associated raw data
- A description of any restrictions on data availability

De novo mutation data of SSC, ASC and MSSNG cohorts are publicly available in the following corresponding papers: SSC (PMID: 25363768), ASC (PMID: 25363760)

Field-specific reporting

Please select the best fit for your research. If you are not sure, read the appropriate sections before making your selection.

☒ Life sciences ☐ Behavioural & social sciences ☐ Ecological, evolutionary & environmental sciences

For a reference copy of the document with all sections, see nature.com/authors/policies/ReportingSummary-flat.pdf

Life sciences study design

All studies must disclose on these points even when the disclosure is negative.

Sample size	Sample size in the SSC and ASC cohort was determined based on the availability of existing data. The samples size of the patient cohort with TANC2 mutations was determined based on the collaborative network. We tried to collect as many patients with TANC2 mutations as we were able. For behavioral analysis in the flies, sample sizes for each group are noted in the graph for each genotype.
Data exclusions	During ASD candidate gene prioritization using the SSC quad families, we excluded LGD mutations with high minor allele frequency because we only focused on the extremely rare de novo LGD variants in this study. We also exclude de novo LGD mutations with low confidence in term of the sequencing quality. The sample size of 15-24 flies in each group using appropriate controls is standard across Drosophila courtship literature.
Replication	The significance for the excess of TANC2 de novo LGD mutations in NDD cohorts were performed by the two statistical models as described above, which were consistent with each other. The Drosophila NMJ phenotypes were measured in two RNAi lines, and was rescued by wild type.
Randomization	N/A - Not relevant to this study.
Blinding	In terms of ASD candidate gene prioritization analysis using the SSC simplex quads, we were not blinded to group allocation during data analysis, as preexisting genetic data published for this cohort make blinding neither possible nor practical.<br>In terms of TANC2 patient collection, investigators were blind to each other regrading collecting/recruiting the patients.<br>In terms of the drosophila NMJ analysis, we were blind to group allocation regarding counting the bouton numbers and measuring the GluRIIA fluorescence. For courtship analysis, users are blinded and analysis is performed in an unbiased and automated fashion.

Reporting for specific materials, systems and methods

Materials & experimental systems

n/a	Involved in the study
☒	☐ Unique biological materials
☐	☒ Antibodies
☒	☐ Eukaryotic cell lines
☒	☐ Palaeontology
☐	☒ Animals and other organisms
☐	☒ Human research participants

Methods

n/a	Involved in the study
☒	☐ ChIP-seq
☒	☐ Flow cytometry
☒	☐ MRI-based neuroimaging

Antibodies

Antibodies used	The primary antibodies we used were anti-GFP conjugated with FITC ab6662 (Abcam), 1:500; anti-Elav (Embryonic lethal abnormal vision) rat monoclonal: 7E8A10(DSHB), 1:200; anti-Repo (mouse monoclonal: 8D12(DSHB), 1:50;rabbit anti-HRP at 1:1000 (code number: 323-005-021; Jackson ImmunoResearch, West Grove, PA);mouse anti-DLG at 1:50 (4F3; DSHB); mouse anti-GluRIIA (8B4D2; 1:50; DSHB).The secondary antibodies used were Alexa 488- cy3- or -647 conjugated anti-mouse and anti-rabbit secondary antibodies at 1:250 or1:500 (Jackson ImmunoResearch, West Grove, PA).
Validation	The primary antibodies are all validated by immunofluorescence staining (Figs. 5-7). Additional validation and peer-reviewed papers are available on the manufacturer websites.

Animals and other organisms

Policy information about studies involving animals; ARRIVE guidelines recommended for reporting animal research

Laboratory animals	Drosophila melanogaster, UAS-rols-RNAi lines - y1sc*v1; P{TRiP.HMC04426}attP40 (BDSC_56986) and y1sc*v1;

Laboratory animals	P{TRiP.HMJ22326} attP40(BDSC_58262), UAS-rols - y1 w*; P{Mae-UAS.6.11}rolsLA00796 (BDSC_22194), rols deficiency line - w1118; Df(3L)ED4475, P{3'.RS5+3.3'}ED4475/TM6C, cu1 Sb1 (BDSC_8069), UAS-mCD8::GFP - w*; P{10XUAS-mCD8::GFP}attP2 (BDSC_32184), UAS-nls::GFP - w1118; P{UAS-GFP.nls}8 (BDSC_4776), G-TRACE - w*; P{UAS-RedStinger}4, P{UAS-FLP.D}JD1, P{Ubi-p63E(FRT.STOP)Stinger}9F6/CyO (BDSC_28280), Repo-Gal4 - w1118; P{GAL4}repo/TM3, Sb1 (BDSC_7415), C57-Gal4 was described57. The newly generated y1 w*; Mi{Trojan-GAL4.1}rolsMI02479-TG4.1/TM3, Sb1 Ser1 (BDSC_76150), y1 w*; Mi{PT-GFSTF.1}rolsMI02479-GFSTF.1 (BDSC_64471), and UAS-TANC2 - y1 w*; PBac{UAS-hTANC2.B}VK37 (BDSC_78452) were generated in the Bellen lab and have been deposited in BDSC. The 80 kb genomic rescue line for rols was generated by Genetivision by insertion of the CH321-52G11 P[acman] genomic BAC clone into the VK37 landing site on chromosome 2.
Wild animals	N/A - This study did not involve wild animals.
Field-collected samples	N/A - The study did not involve samples collected from the field.

Human research participants

Policy information about studies involving human research participants

Population characteristics	In total, 19 ASD or ID/DD probands (12 male, 7 female, ages 4-40) with TANC2 mutations (LGD mutation, microdeletion) and five patients with TANC2 de novo missense mutations were collected from different collaborative centers or the literature. Parents and affected siblings of some of the probands were also included in this work.
Recruitment	The participating patients/families with TANC2 mutations were recruited at the collaborative centers with the purpose of genetic etiology research and/or diagnosis. The collaborative centers were blind to each other regrading collecting/recruiting the patients.
