# Supplementary material for: Disruptive mutations in TANC2 define a neurodevelopmental syndrome associated with psychiatric disorders
Source: Nat Commun. 2019 Oct 15;10:4679. doi: 10.1038/s41467-019-12435-8 (PMC6794285; doi:10.1038/s41467-019-12435-8)
Supplement: Supplementary file 7 — Description of Additional Supplementary Files [file 41467_2019_12435_MOESM7_ESM.docx]

**Title:** Supplementary Data 1
**Description:** Curated de novo LGD mutations in SSC simplex quads.

**Title:** Supplementary Data 2
**Description:** De novo LGD mutations of the 58 prioritized genes in SSC and ASC cohorts.

**Title:** Supplementary Data 3
**Description:** All rare amino acid changing mutations detected by smMIP sequencing.

**Title:** Supplementary Data 4
**Description:** Detailed clinical information of patients with TANC2 disruptive variants or de novo missense variants.
